# Proceedings of the American Dental Association

**Published:** 1870-10

**Authors:** 


### ARTICLE III.

### *Proceedings of the American Dental Association.*

(Concluded.)

Dr. Kulp. It was always his opinion that a mother could give her children good teeth. He cited a case where a lady had had several children with bad teeth. But seven years ago he had taken her under treatment, and since that time she had had three children born, who were in better condition with regard to teeth.

Dr. Crouse would like to know the condition of the mother with the first and second child.

Prof. Stellwagen. Does the gentleman mean physiologically ? Worse with the second than the first.

Dr. Crouse thought there was little difference whether bolted or unbolted flour was used. He thought cow's milk could not be too rich and did not need any additional water for food for infants.

Prof. Stellwagen. What is the proportion of water in cow's and human milk ? Is it not greater in the latter ? and will not the former be too rich for their delicate digestive apparatus ?

Dr. Crouse could not say, but he thought pure milk none to good for babes.

Dr. Walker. We have had light, and good light. I believe the time will come when children will be raised with good teeth. I think local habits have more to do with it than climates. Scrofula has been spoken of,—where do we get our word scrofula ? It is from scrofa, a sow ; this is why the negroes have worse teeth than the whites. The people spoken of with good teeth are free from this disease.

Prof Stellwagen. Do the Israelites have better teeth than Christians or those who eat pork ?

Dr. Walker. Yes ; and this is why the God of Abraham forbade the use of pork.

Prof. Stellwagen. The gentleman's experience is very different from mine. Some of the worst teeth I have ever had under treatment were in the mouths of strict Israelites.

Dr. Peebles' practice is among many of the Israelites ; they have worse teeth but do not take so much care of them. He had come in contact with the Indians also, and the young Indian had bad teeth. The exhumed skulls of Indians examined also had defective teeth. Those living at the time examined may have fed upon fine flour, but the exhumed could not have had bolted flour. With the negroes he found they had better front teeth, but worse molars.

Dr. Forbes. It has for many years been asserted that the teeth of Americans are far inferior to those of Europeans; he would take exceptions to this. He at one time had examined the mouths of aborigines; he had learned from one old lady who had had much experience with Indians; she said some tribes had worse than others. He thought Europeans had worse teeth than Americans. He had the report of a German lady that had examined the teeth of her relatives in Germany and they were worse than hers. Another lady had asked him why American dentists were the best, if it was not from the demand for them; and in answer he told her that she was not educated to know how bad her teeth were. He was proud to say that the American women were so much better looking, especially about the mouth. In Europe a lady would be considered disgraced to have it known that a tooth was filled. He considered it far more reasonable to have a tooth jeweled than the ears or nose. As a rule, the Irish in their own country have better teeth than the Americans. But they generally drop out at forty. It had been said that it was criminal to extract a tooth under any circumstances. He would not assent to this,—it was the most consummate falsehood. How about treating teeth and not losing one? He had suffered from operations by those who claimed never to have lost a tooth, and now had but few left.

Dr. Cobb thought that Dr. Allen's paper, with regard to food, was instructive; but how could we practice what he advises? He feared we did not live as we ought to live; he comes with an instructive paper, but does not live as he advises. He wants to see people practice this. He thought it the duty of the dentist to attend to the hard structures and let the physicians attend to the others.

Dr. Allen meant that in early life he did not use brown bread, but now he does always, and his children have never had a tooth extracted, so far as he knows. He lost his teeth from sickness and ignorance in the abuse of calomel.

Dr Walker thought the time would come when extracting teeth would be a crime.

Dr. Dennis. An epitaph was brought to his mind by these discusssons—

"I was well, wished to be better, and here I am."

We will adjourn to the tea table and eat as before. Consistency is a jewel, but it is as as rare as the most precious.

Prof. Taft. Dental chemistry is simply vital chemistry. This subject is broad, and the overlapping of these subjects, vital chemistry, physiology, and pathology, has been the cause of the rambling. He took exception to the remarks of Prof. Cutler, with reference to crowding the system with one kind of food and depriving it of another. At all times during life the phosphate of lime is required; he does not believe that the system will take up more than required. That the bones will be solidified by an increased amount of mineral material in the food, he thinks a fallacious doctrine.

Prof. Bogue. There is one point that may be brought in, that of discoloration of dental tissues. This may be due to hæmatin, iron, or one or both, if it exists within the dentinal tubuli. He conceived it due to oozing of the contents of the blood globules into the dentinal tubuli.

Dr. Forbes thought the salubrity of the Irish climate was most perfect, and he wondered they did not live forever. The meat took the place of bread; in many cases, when used as food, furnished the required amount of mineral substances.

Adjourned.

THIRD DAY.—*Morning Session.*

The meeting was called to order at 10.05 A. M., August 4, by the President. After prayer, the minutes were read and approved.

Dr. Cushing, Chairman of the Committee on Operative Dentistry, read a very able report, which noticed the recent improvements in this branch, praising the rubber dam, and the generosity of Dr. Barnum, in giving the result of his invention to the profession,—reviewing the subject of adhesive gold, and the use of heavy foils, which it favored; finally calling the attention of the profession to some of the labor-saving machines for excavating, filling and finishing.

Prof. Knapp then read two papers :—"The Saving of Time in Dental Operations," and "Cylinders—the Method of Making and Using them."

He recommended the operator to provide himself, for making cylinders, with a desk having a velvet top, a box divided into small compartments for holding different sizes of cylinders, an ivory paper-folder, a Swiss broach, upon which to roll the gold, the end being broken off and sharpened, and a pair of foil shears.

He had used many numbers of gold, from 2 to 30, but preferred Nos. 4, 5, and 6. When very large and long cylinders are needed, an entire sheet of No. 4 may be used; but for smaller, the sheet is cut into strips before folding.

To fold into strips or tapes, he first lays the piece of gold of the size required upon the velvet-covered top of an ordinary writing-desk, and then indents with the edge of the paper-folder, and folds gently. The tape is then placed on the inside of the forefinger of the left hand, the hand being turned with the palm up, and the end of the strip almost to the end of the finger. The broach is taken up by the right hand and laid upon this end of the tape, and the gold rolled upon it by closing down the thumb of the left hand and drawing it backward toward the first joint of the forefinger. The rolling of the cylinder is completed by twisting the broach, thus once started, with the thumb and forefinger of the right hand. For very short cylinders, the tapes may be made by folding the sheet of gold and then cutting into very narrow strips with the foil shears. When the rolling of the cylinder has been completed, a few more turns may be given with the nail of the left thumb pressed against one end of the cylinder.

He claimed that the method described originated with Dr John H. Clark, of New Orleans, and referred to the *American Journal of Dental Science*, vol. i. p. 23.

Some cylinders may be made larger at one end than the other, by folding the tape so that one of the sides is the thicker. For filling pulp canals, the breadth of the tape is made almost equal to the length of the canal, and one end is folded to within a short distance of the other; then the end which has been made by the doubling is carried nearly to the end first folded down. This doubling up of one end is continued until the length of the canal to be filled is obtained and the gold presents a series of slight steps, the first being the thickness of the tape, the second double, the third quadruple, the fourth eight times, and so on, duplicating. The broach is then put on at right angles to these folds, and the whole is rolled into a tapering horn with the smooth sheet of tape outside and the doubled steps inside.

The method of filling consists in closing up the crevices by small cylinders stood on end, and then arranging others parallel with these around the walls, so that the ends project out about one-third of the whole length of the cylinder. They are flattened and partly condensed against the walls by lateral pressure. Others are then placed around within these aud similarly worked. The largest are rolled soft and used first, while the small hard ones are for finishing, keying all together in the small holes pierced by longitudinal pressure on the cylinders with wedge-pointed instruments. He spoke of the three edged hatchets and hoes he uses for excavating, and the wedge-pointed pluggers for filling with cylinders.

A paper by Prof. Frank Abbott, of New York City, "Light versus Heavy Gold Foil and Crystal Gold," was read.

Prof. Knapp. The paper just read advocates invariably the use of soft gold. The perfection of the filling, whether it be hard or soft gold, depends most upon the manner in which it is introduced.

Dr. Peebles. Two facts are called to our attention by the reading of these papers. Prof. Knapp gives the credit of introducing cylinder fillings to Dr. Clark, in 1850. Dr. Spalding, of St. Louis, once claimed priority, and Dr. Ide, of Logan (now Columbus), Ohio, in 1839, had filled with cylinders, and recommended it to be done as cigars are put in a tumbler. Dr. Parmly, of New York, when in Louisville, over forty years ago, had gold rolled, as he could not obtain foil, and used it for filling teeth. He wished these facts on record, since the use of the mallet was mentioned in Fitch's work on dentistry, long before it was introduced as original by another.

Dr. Carroll criticised the use of the terms "cohesion" and "adhesion." He understood the former to mean the attraction of the particles of any substance for each other.

Dr. Morgan believed Dr. Clark had the honor of introducing cylinder fillings, but he had examined some made in 1832 of cylinders. Dr Badger claimed to have shown Dr. Clark the first cylinder filling. A gentleman here (Nashville) had a filling inserted in this way at the age of sixteen, and is fifty-four years old now.

Prof. Knapp gave Dr. Badger the credit of using cylinders, but the doctor admitted that he did not make the entire plug of them; he forcd them into plugs on finishing.

Dr. Morgan. I have seen Badger operate, but never saw him use a strip.

Dr. Fouché said his preceptor, Dr. G. P. Norman, of Cincinnati, used cylinders in his presence in 1847–8.

Dr. Dickerman. Dr. Flagg instructed me, in 1842, how to make and use cylinders.

Dr. Walker. The same idea so often occurs to active minds that it makes but little difference who made the first cylinder. He found that he must have instruments with points to go over and over again every part of the cavity. Where a cavity is irregular, he tries to make it simple. On the approximal surfaces of the bicuspids he can get the best fillings with cylinders, but where there are irregular walls he uses soft foil or adhesive gold and mallets.

Dr. McKellops. The point to gain is that Dr. Clark was the first to put before the profession a description of how to make and use cylinders for filling teeth.

Prof. Bogue said: Under the head of Operative Dentistry we may consider some points—how our operations may be made neater by keeping our fingers and nails clean, by avoiding the use of them in our hair, etc.; napkins should be kept in order, instruments clean and polished. He said Dr. Carroll had criticised the word "cohesion" meaning sticking "with,"—"adhesion," sticking "to" any substance. Criticising the nomenclature of the profession, he thought we should drop "eye tooth" for "cuspid," which is better. "Tartar" is an incorrect term when applied to "salivary calculus." We often hear "destoying nerve," "taking nerve out," etc. spoken of, meaning "taking out *pulp.*" It is not to be doubted that cylinders will make a most perfect filling in many cases, and the microscope will demonstrate that. There are, however, some cases where crystal foil or gold is needed. *Teeth must be filled with judgement as well as gold.* He would no more abandon cylinder fillings than rubber dam. We must use all kinds, as the case requires.

Dr. Carroll thanked Prof. Bogue for the opportunity with regard to the criticisms of nomenclature. He differed on the Latin prefixes "co" and "ad." "Co" he defines "with." "*Ad*" means "to." Now, soft foil is always cohesive, but is not adhesive.

Dr. Allport. I hold in one hand a letter with a stamp adhering to it; in my other hand is a fan,—the particles of the handle cohere.

Dr. Dennis. Adhesion is a union of unlike substances; cohesion the union of like substances.

Dr. Dean. Cohesive attraction holds together like particles of matter, or atoms of the same kind. Adhesive attraction exists between unlike atoms or particles when in contact with each other.

Dr. Mills eulogised the paper of Dr. Cushing, of Chicago. It was an important matter that we should pursue our practice without prejudice, and simply for the truth. We should spend more time, and examine closer into the preparation of the cavity for filling. He would as soon think of abandoning the rubber dam as to attempt to fill with cylinders, but has been much pleased with the views advanced. A remark was made by Prof. Knapp that he wanted to fill a difficult cavity, not a simple one. He thought much could be learned from simple cavities. The principles undelying filling must be studied, and difficult cavities reduced to simple ones. With reference to the number of instruments. He has used many to learn, and he thinks here is another governing principle. He has come dowh to a few, which he finds valuable. Varney has presented thirteen; he takes out four and gets along just as well as well as he did with all. His nervous temperament makes him try many He agrees with Dr. Knapp as to the form of cavities,—the walls almost perpendicular.

The Recording Secretary read an invitation to visit the Tennessee Hospital for the Insane.

The invitation was accepted, with the thanks of the Society.

Dr. Crouse moved for an evening or a night session, to hold the election for officers and to determine the next place of meeting. Carried.

THIRD DAY.—*Afternoon Session.*

Committee of Arrangements reported list of new members and names of the members for the different committees.

Dr. Corydon Palmer took the floor, and stated that his subject was in continuation of that treated of last year. He desired to show how everything pertaining to the mouth may be made of use in practice. Every fissure in a tooth filled should be marked and the charge made immediately after the petient has been under the hands of the operator; they are both better able to judge of the amount of force expended, etc. He divided the mouth into four divisions.

• First, he exhibited a drawing of a head divided into four by a perpendicular and transverse line; this gave an upper and lower right and left division. In making a record it is well to know how to note this,—L. Sup., L. Inf., R Sup., R. Inf. divisions.

Second drawing gave the teeth, thirty-two in number. Commencing at central incisor, he numbers backward to eight; in this way he has the teeth of the four divisions described by numbers; thus, the lateral incisor is No. 2, cuspid, 3, and so on to wisdom tooth, No. 8. The teeth thus become better known by their numbers than by their names. By the shape of the mark made he knows the kind of filling and the position on the tooth by its relative position on the number designating the tooth.

Almost every dentist has some sort of short-hand or way of abbreviating a description of the operation. The time to make these marks or memoranda is immediatly after completing the operation. Demonstrations then followed. He said that he was sorry to see so much time wasted in making pellets, cylinders, etc. They are filled with animal matter from the hands; while pressing and squeezing them in, spring the cavity in the tooth, and cause a constant antagonism between the attempt of fillng to escape and the tooth to retain it. Since 1860 it has been demonstrated by our prophet that a tooth can be built up, with all its cusps and fissures; yet there are some who will yet crowd gold into a cavity. No first-class operations can be made if the gold is handled with the fingers. In using soft foil, it don't make so much difference how you fill, so long as you can put it off; but in ten years or so take it out and it is offensive, and the animal matter in it will burn.

You need not fear cutting down crown surfaces or fissures, for with adhesive gold this may all be replaced. Make the cavity thoroughly open, and put a mat upon the bottom; then build up packing toward the walls. This is first-class, and anything short of it is short of the highest attainment in our science. It has been stated here to-day that the cylinders make the most perfect and solid fillings. It is not true, —they will not do. Begin and build up solid from the bottom. Do not object to the heavy foils, but with No. 4, if you have proper instruments and operate correctly, you will obtain good results.

You must understand how to have your instruments right, and encourage our manufacturers. What is one or two dollars for an instrument? The difference of a line will make an instument unfit for use. Just pay them to put the talent in it; have the serrations fine. It is most essential to have a fine point. Don't put the instrument on with a nervous force or jerk, but steadily, and press it down, holding it tight; then with a lead mallet strike a following blow, and let it not spring off; this vibration is what hurts the tooth. You strike with a steel mallet, or wood, or bone, etc., and bounce it off, but with the lead you hold the instrument until after the blow dies away.

In a recent case of atrophy with ridges and pits, I used the rubber dam and immediate wedging; had an 8-oz. mallet and very light instrument. The instrument should be in length in proportion to its size, and all according to what you may wish to do. With regard to mallets, I have a band of iron made, united with hard solder, and run the lead into it for a head. The making of the gold foil is confined to the manufacturer. We know there is a difference between adhesive and soft foil. I want that with a beautiful, bright, metallic surface. One-third of the value of gold is destroyed by pressing it in a screw-press, leaving the mark of the paper upon it. To use it I cut the foil, let it fall upon a napkin and roll it up with that. This roll is cut in pieces or attached at one end, and then built around. For annealing, the tube of the lamp should be made of platina or glass, so as to have a flame free from oxides. Be careful of the match, as the phosphorus may injure the gold.

I will describe how I filled a left superior lateral incisor, where one-fourth of the labial wall was cut away to get at the cavity. Get a perfectly smooth and straight labial wall; do not bevel it at all. This is done to have it strong. It may be started with a small chisel,—let it be strong and sharp. Then use a file to prepare the edges; get margin or edges all right; proceed until the cavity is fully prepared; with a fine drill, make a small retaining-point, or make little grooves. It is astonishing how much little pits will help to retain the gold. The rubber dam is indispensable; you will come short of making a first-class operation if you do not use it. Put little hooks in the corners of the rubber, and attach to strings carried over the ears, to hold the dam up out of the way. He said that he divided the finishing of a filing into six stages:—first, filing; second, scraping; third, stoning; fourth polishing, with fine pumice; fifth, polishing with fine chalk; sixth, burnishing. He cautions all to be sure and wash off the filling well with soapsuds before burnishing.

THIRD DAY.—*Evening session.*

After much discussion, Atlanta, Ga., was chosen, upon the third ballot, for the next place of meeting; and the association then went into the election of officers, with the following result:

*President.*—Dr. W. H. Morgan, Nashville, Tenn.

*First Vice President.*—Dr. S. W. Dennis, San Francisco, Cal.

*Second Vice-President.*—Dr. J. R. Walker, New Orleans, La.

*Corresponding Secretary.*—Prof. I. A. Salmon, Boston Mass.

*Recording Secretary.*—Dr. M. S. Dean, Chicago Ill.

*Treasurer.*—Dr. W. H. Goddard, Louisville, Ky.

*Executive Committee. For three years.*—Dr. G. H. Cushing, Chicago, Ill.; Prof. J. S. Knapp, New Orleans, La.; Prof. J. Taft, Cincinnatti, O. *For two years.*—Dr. W. W. Allport, Chicago, Ill.; Prof. E. A. Bogue, New York, N. Y.; Dr. I. Forbes, St. Louis, Mo. *For one year.*—Dr. S. J. Cobb, Nashville, Tenn.; Dr. A. C. Ford, Atlanta, Ga.; Dr. J. P. H. Brown, Augusta, Ga.

*Committee on Publications.*—Drs. M. S. Dean, W. W. Allport and G. H. Cushing.

*Committee on Dental Physiology.*—Profs. Homer Judd, Thom. C. Stellwagen, and Dr L. Augspath.

*Committee on Dental Chemistry.*—Prof. T. L. Buckingham, Drs. C. C. Carroll and J. G. Willis.

*Committee on Pathology and Surgery.*—Prof. W. H. Atkinson, Drs. J. R. Walker and W. H. Morgan.

*Committee on Operative Dentistry.*—Prof. J. S. Knapp, Drs. H. J. B. McKellops and P. G. C. Hunt.

*Committee on Mechanical Dentistry.*—Drs. John Allen, W. H. Eames, S. B. Palmer, C. M. Wright, and B. B. Alford.

*Committee on Dental Education.*—Drs. G. A. Mills, S. J. Cobb, and, Prof. L. D. Shepard.

*Committee on Dental Literature.*—Drs. G. H. Cushing, R. N. Lawrence, and J. N. Crouse.

*Committee on Voluntary Essays.*— Drs. S. H. Beard, and C. D. Cook.

*Committee on Dental Histology.*—Dr. W. W. Allport, and Prof. J. H. McQuillen.

*Committee on Dental Therapeutics.*—Dr. H. S. Chase, Prof. E. A. Bogue, Drs. Thos. T. Moore and C. P. Baird.

*Committee on Dental Instruments and Appliances.*—Dr. J. B. Morrison, Prof. I. A. Salmon, Drs. S. W. Dennis and Corydon Palmer.

*Committtee on Prize Essays.*—Drs. I. Forbes, J. P. H. Brown, W. C. Dyer, A. C. Ford, and J. W. Moffit.

*Committee on Dental Ætiology.*—Profs. J. Taft, S. P. Cutler, Drs. R. W. Varney and W. O. Kulp.

FOURTH DAY.—*Morning Session*

Called to order at 10 A. M.

The session was opened with prayer. Minutes read and adopted.

An amendment to the constitution, offered by Prof. Buckingham, last year, was then read. It provided that

"No person shall be a member of this association who holds a dental patent, or is or shall be interested in one."

After considerable debate it was lost.

Dr. Dennis called for Dr. Mills to continue his remarks.

Dr. Mills thinks the best excavators are the three-edged; he received from Dr. Brockway, of Brooklyn, N. Y., an instrument that is a sort of scoop; this is very generally useful; it does away with burs. His manner of filling teeth is to wedge immediately. The main stepping-stone to success is the preparation of the cavity. He uses two excavators, a scoop and a file, to finish edges. Like Dr. Palmer, he wants perpendicular walls. He has used many kinds of gold, but now prefers adhesive. He sometimes uses Morgan's plastic gold, in the bottom of the cavity, to facilitate and hasten operations. He uses foil, mostly Nos. 8, 10, 15, 40, 60, and 120, prefering Nos. 15, 40, and 120. He makes slots for retaining-points, and commences with heavy num bers, as they retain better and stronger. The heavy foils, he thinks, produce a better class of work, but require more care in packing. He thinks that if fillings were hollow, but the walls well adjusted, they would be better. The packing of gold around the surfaces and margins of the cavity, he thinks well of. Sometimes, in turning over the heavy foil, or folding, it makes a corner, and some object to it. He practices burnishing or beating the edges of the tooth and the filling, bending down solidly. When he says "beat," he does not mean a heavy or brutal hand,—it can be done with heavy pressure. In using the mallet, it should give the blow in the direction of the axis of the tooth. he has had cases where the mallet could not be used.

Prof. Knapp is no advocate of any extensive use of cements, but believes they may do well in some cases. He would not call attention to anything but a wash for cement; it is the *Liquor Sodæ Chlorinatæ.* It is a deodorizer and alkaline, quickly removing all odor or smell from the hands. It gets rid of the oxide from cements. Equal parts of water with Labarraque's solution first, and finally washing repeatedly with alcohol, cleans the amalgam very perfectly.

Dr. Dickerman, in bleaching teeth, removes all of the carious portion of the tooth, and dresses with camphorated spirits for two or three weeks. If it is advisable, lines the cavity with oxychloride of zinc. A tooth is not dead so long as it has any attachment, and it may be restored to usefulness even when this is very slight. Abscesses that have been discharging from one to twenty-seven years have healed. We then find cartilage formed. With regard to gold, he had seen teeth filled with from No. 2 to 640, or No. 20 doubled 32 times. Good fillings may be made with all numbers.

Dr. Mill. Gentlemen seem to have lost sight of principle when they talk of leaving a vacuum. There can be no vacuum in natnre. He does not approve of sponge gold shingled over. With relation to cement, there may be circumstances where it may be of use, but it must be with caution. Many may jump at it, as they have not been able to fill with gold. He has in his own mouth the eleventh filling of a student, which is as good as any body could desire. All, or most all, can, by proper education soon learn. He thought it would be well to select a few of the best operators and pay them to go over the United States and teach dentists how to operate. Talk of teaching people,—let us teach ourselves until we are able to know what we are about. Why is the medical profession not willing to acknowledge us? It is because we are not educated.

Dr. Forbes. With regard to the number of instruments, as we advance, very few are used. The dental manufacturers, selling so many, reminds him of a teacher selling his instruments to his student, when he knows that the poorer the operator the more he needs good instruments. It is an axiom, that a man does not know a thing if he cannot communicate it to others. One operator says we can learn more by witnessing a single operation than hearing it spoken of for years. He was once taught here that napkins were not necessary and that *debris* can be blown out of a cavity! He thought a tooth could be better built up by cylinders than any other kind of pellets. In filling a tooth, Dr. Palmer made a slot and put a piece in it, holding it in its position until condensed. He then built down, claiming that, in twenty years after, if taken out, it would be pure and clean beneath! Now, if the heat beneath is 95°, what if the patient eats ice? It will reduce it to 35° or 40° and must contract.

We have just heard that Prof. Abbott's experiments are of no consequence!

If a cylinder were placed at the side of a cavity and condensed there, the filling may be thus one-half completed, and the cavity is gradually reduced as more and more are added. Cannot the small cavity left be filled as well as a large one? A cylinder filling will remain perfect as long as a keystone in an arch!

The paper by Prof. Knapp reads as if all should be made by cylinders with hand-pressure. If the mallet is used, it would be found to be a labor-saving machine.

He never touches his fingers to the flesh, but always lays a napkin in the mouth. Operators are telling us that by heavy foil they can put in more gold. He would like the experiment tried with cylinders.

With regard to operations in St. Louis, they have a book like a bill book with two diagrams of the mouth on each page, so that one can be torn off and sent to the patient. Then, by abbreviations by letters, they make the charges quite as well as by complicated, arbitrary signs.

Dr. Walker has met with success with biborate of soda for washing amalgams. He presented as an alloy for casting metal plates, in place of vulcanite—

Bismuth, 1 part;
Pure tin, 15 to 20 parts.

To make sharp castings, there is nothing that he knows of better.

Prof. Atkinson. The only way to learn is by close attention and presence at operations. He would prefer to have the filling hollow, if solidly adapted to the walls. With few instruments, under the inspiration of necessity, we can make excellent operations. We should not talk of contraction of gold from cold, and forget the tooth substance. There is no appreciable change of the two, or if any, it is not of importance.

How many are here who never saw their preceptors put a filling in a tooth in the mouth! Do not operate for any one who will not permit a third party to be present. Let the students excavate teeth in the laboratory, and finally go to the office and do *as some of my students have done,—never make a failure.*

It is damnable for a man to wilfully destroy a pulp at any time. If any vitality is left, there is hope of saving the organ. Twenty years ago I came to that conclusion. November 4, 1857, I dedicated myself to save each, all, and every part it was possible to do.

Prof. Stellwagen exhibited two pairs of excising root forceps made according to patterns described by him in the Dental Cosmos for September, 1869. He wished that the members would suggest any improvements they might think of. He considered that, when roots are broken off below the alveolus, these are invaluable instruments, as they insure the extraction of the remaining portion with but little crushing or grinding of the surrounding tissues, making clean cuts through, instead of tearing out the sockets with the roots.

He also called the attention of the members to a pair of metallic boxes like impression-cups without handles, in which softened gutta-percha could be placed, and the whole pressed upon the teeth to hold them in position in case of fracture By means of holes through the plates, he secured the washing of the mouth with jets of water, and through an opening in the front, fed his patient, and also kept the mesian line of the mouth in sight. These splints could be thus used for different cases, and admitted of being quickly adjusted. They were modifications of Dr. Allport's and Dr. Gunning's splints, differing from the latter in having the plates wired instead of soldered together, thus admiting of perfect adjustment of the two maxillæ without removing the splint. Changes, if desired, could be effected by drawing some of the wires tighter and loosening others. A few sets of these would prepare one for almost any case of fracture, and he thought, instead of plates, mere rims united by transverse bands would do better. The pair exhibited had been used by him in a case of fracture of the inferior maxilla, and a perfect occlusion of the teeth was obtained in a short time, where it had seemed at first to be very nearly impossible to hold the fractured ends in position.

Dr. Walker urged the importance of endeavoring to instruct a patient to keep fillings upon the approximal surfaces of the teeth clean.

Dr Palmer. A cement in hardening will shrink a little; it is said of amalgam that it will enlarge a little. The difference between a filling which hardens after putting in and a metal one that is hardened by pressure is very great. He don't advocate amalgams, only as experimental. Pure gold and pure tin are the very best materials used. If the gold don't come in contact with the cavity, a change takes place. Emery-paper is too harsh for use in polishing.

The report of the committee on Mechanical Dentistry was read by Dr. S. B. Palmer, chairman. It called for a substitute for vulcanite.

Dr. Allport took exceptions to *cheap* dentistry.

Report of Committee on Dental Education, by Dr. M. S. Dean, and accepted.

Dr. Cobb, of the same committee, read a paper recommending a dental journal to be published with the proceeds of a fund established for the purpose from the treasury of this society

Dr. Allen could not help comparing the condition of the dental profession with what it was when he studied. We have had many advances,—colleges, societies, journals, works, etc. While all this is advancement, we still want more, and it is the increased light that brings us to wish for greater perfection. The most of the artificial work made to-day will not compare with what it was twenty years ago. He feared we were neglecting this and attending only to operative dentistry. He gloried in the improvements in operative dentistry, but he regretted to see the association ready to pass over mechanical dentistry.

Prof. Taft. Dental education should interest all. Shall those who come after us be equal to or superior to us? We should prepare them to meet the exigencies of an age when one revolution is rapidly following in the footsteps of another. There are other features. Are all the men now in the profession prepared for it? As we are laboring to raise them, let us consider if there is nothing more to be done than there is now. We have our periodicals and our associations: these reach but few. Of the eighty associations, perhaps one thousand would count all the members that are in them. Can nothing be proposed by which to reach the balance of the profession? This association had once attempted, by appointing a committee, to aid in organizing local societies; through it many were formed. This committee has died out. It should not be so; there is yet a large field for the work. Another idea is to appoint definitely a committee to go out among the profession, and gather them in classes to teach them and stimulate them to learn more, making normal classes, supported by this association or other means. Many have already offered to assist in such an undertaking. This would habituate dentists to meet together, and they would soon support the classes themselves. His great desire is that the most should be done to elevate the profession that our resources will admit of.

Dr. S. B. Palmer agrees with those who would improve our science and disseminate a general knowledge of it. He has given the matter much attention, and among the means offered there might be found some objectional feature. He proposed the printing of a circular or card, enjoining the importance of cleaning the teeth, which may be free from advertisements, and obtainable from this association at a moderate cost by members for distribution among their patients.

Dr. Crouse. Let us make better practitioners of ourselves, so as to show the people the greatest difference between the true and false dentist. He had suffered from his office and college instruction. If it had not been for dental societies he would, no doubt, have been plodding along yet. A deficiency of colleges is that they have not enough clinical demonstrators, making the clinics or the infirmaries the great thing. Men who are run away with by their hobbies are not fit to teach! In dental societies he first discovered his deficiencies, and this had stimulated him to improve himself.

Prof. I. A. Salmon moved to read all reports first, and afterward to have the discussion in regular order. Carried.

Dr. M. S. Dean, Chairman of the Committee on Publication, reported that four hundred and twenty copies of the proceedings had been printed, at an expense of $380, and the price to those not members was fixed at $1.25 each. Two hundred and fifty copies of the new constitution had also been printed.

Dr. T. T. Moore offered, as an amendment to the constitution, that no one shall hereafter become a member who has a dental patent, or is interested in one. Laid over, according to law, for the next annual meeting.

Dr. Forbes offered the following:

*Resolved*, That the members are requested to bring with them to the next annual meeting such pluggers and excavators as they have in general use, so that when presented to the Committee on Instruments the committee may be enabled to make up a *complete set*, containing no more instruments than are necessary.

Dr. Shadoan spoke of the dissatisfaction at the place of meeting chosen. He moved to reconsider the question, which resulted in the selection of Niagara.

FOURTH DAY.—*Afternoon Session.*

Called to order.

Dr. Goddard offered, as an amendment to the constitution, that the meetings be held annually at such *time* and place as may be designated. Laid over for the next meeting

The minutes were then read.

Greenbrier, White Sulphur Springs, Virginia, was then chosen as the place of next meeting, as a compromise between Niagara, Atlanta, and San Francisco. The announcement of the result was received with great satisfaction, and a question which had disturbed the harmony of feeling was thus ended.

Committee on Dental Ethics, Dr. Morgan, Chairman, reported with regard to the case of Dr. J. A. McClelland, finding him guilty on his own admission, but with mitigating circumstances, and moved that he be allowed five minutes to be heard. The subject was then, on motion of Dr. Cushing, postponed until Dr. McClelland should be present.

Dr. Kulp moved that a committee of three be appointed, who shall prepare a circular upon popular education, and furnish the profession copies for distribution, at a rate sufficient to cover expenses of publication. Adopted.

Drs. Kulp, Walker, and Peebles were appointed.

Dr. McClelland, of Louisville, then came in and made an explanation.

Prof. Atkinson moved that it be deemed satisfactory. Carried.

The customary ceremonies and speeches incident to installing the new officers were then gone through.

Dr. Cushing received permission to read a letter from Dr. J. W. Clowes, with regard to the rubber dam.

The writer stated that on the 13th of May, 1864, he was informed by Dr. S. C. Barnum of the discovery of this means of keeping teeth dry to facilitate the dressing or filling of the same. He then proceeded to describe how Dr. Barnum had caused the discovery to be presented to the profession, and how, when brought to the notice of practitioners in various places, it was first declared impossible to apply it successfully, then ridiculed, afterward objected to, and finally very generally adopted.

The Corresponding Secretary was then directed to forward a resolution offered by Dr. Dickerman, in which the American Dental Association formally acknowledged the discovery, and the admiration of the spirit which prompted Dr. Barnum to make such an important invention a free offering to his fellows.

Dr. Goddard wished the coffers of the Association could be well filled, so as to be able to make some appropriation for such cases as this. He would be happy if they had many thousands of dollars,—one of which he would like to see appropriated to the purchase of a suitable microscopical apparatus, so that Prof. McQuillen, or some such person, might give lectures and demonstrations.

The Corresponding Secretary was then directed to forward a resolution of this association acknowledging the gift.

Dr. McKellops. There was a motion here to-day about patents. Here was a young man Dr. Barnum, poor and struggling,—now what encouragement has he for his present to the profession which would have been a fortune to him, if he had chosen selfishly to patent it?

Dr. Allport was in favor of more than the mere motion. He would favor the taxing or voluntary contribution to raise a gold medal from the association.

Dr. Dickerman moved that the members of this association be assessed to the amount of $1000.

Dr. Chisholm suggested that it be by *pro rata.*

Dr. Dennis would like to see it voluntary.

Dr. Crouse would prefer to see this done without compulsion.

Dr. Forbes moved that, in consideration of the great merits of his gift, this association contribute the sum of $1000 to Dr. Barnum.

Dr. Cobb opposed a precedent of this kind.

A member thought this just the thing we did want as a precedent.

Dr. Forbes' $1000 motion was then carried.

Drs. McKellops and Arthur were appointed a committee to get up a gold medal, at their own expense, for presentation by the association to Dr. Barnum.

Prof. Atkinson moved that the Publication Committee be authorized to make the best arrangements with the dental journals for the publication of the transactions of this body. Carried.

Dr. Shadoan moved that a delegate be appointed to the Southern Dental Association. Carried.

Dr. J. A. McClelland resigned.

"Operative Dentistry" was then taken up.

Prof. Atkinson. How would I bleach teeth? If iron discolors, cut all away but as small a portion as possible for strength; fill full of oxalic acid crystals; put a drop of water in the cavity, and mash the crystals in it. There are other agents,—salts of copper, etc.,—if we sufficiently understand chemisty, that would be available to bleach. We can use chlorine gas through a small glass pipette; must be carefully used, as it might cause œdema of glottis. Here the rubber dam is valuable. Chloride of lime, Labarraque's solution etc., are all good.

You must not hope too much in bleaching teeth. The great majority will bleach if left to the open air. Fill with wax for a short time, and, on taking it out, wash with salt water. Pure oxide of zinc with chloride of zinc is good to put in a creamy mortar, filling the tooth full, twirling a piece of cotton in it; then cut out all but a small, thin stratum, to see the color. Don't get the teeth too white.

I generate chlorine gas by black oxide of manganese and hydrochloric acid. This works very rapidly.

The agents supposed to color the teeth may be iron from hematin, as the mineral, or they may be of vegetable origin; if the latter, these colors are bleached by chlorine.

My process for saving nerves, or portions of them, is to cut away all diseased parts of the tooth possible without giving too great pain, and to sop with creosote every day—better twice a day. Often a little pus will come away. I have found the bulb come away, and leaving the pulps alive and red in the roots. In one case I have taken out the pulp of all but one buccal root. When recently exposed I treat at once; but, if not, I treat several days, and then I claim that I am no worse off than at first if I should find the pulps dead. Never put arsenic in a human being's mouth. Then put oxychloride of zinc, of creamlike consistency—introduce on cotton, trimming off and filling with gold; or fill temporarily or preparatively with oxychloride of zinc.

I make this distinction between creosote and carbolic acid. The latter is crystallizable acid from coal; creosote is a combination of two acids—cresylic acid and carbolic acid. Pure Scotch or German creosote from beech wood I prefer, but don't know why; the perception has not got into me yet.

Every diseased action is below the standard of life, none above. I use creosote to make a carbolate and cressalate of albumen, to make a protection like a scab. This I call calciferous matter or it may be secondary dentine substance. I have only a little or crescent-shaped particle form upon one side of the exposed portion of the pulp, and sometimes have seen all filled up. I always leave a little creosote in every cavity, and do not wipe out too dry. I once put in the capping without pain, on the pulp, and there was none for some time after, but the patient returned, suffering, and I opened and found an empty pulp chamber. This is better than destroying nerves any other way. Creosote cannot destroy beyond a certain point, unless the pulp is unhealthy, for the saturated condition of the pulp will not take up more than to make this pellicle, which protects the balance.

Alveolar abscess will not occur, unless the liver or the pancreas is out of order. Lower the circulation, give an emetic, and follow up with a cathartic. To the point or seat of pain in the gum apply hot or cold water; no luke-warm. Hot is above 112°,—say 150° or 160°. Apply hot or cold, which ever comforts the patient. You may begin with hot flannels (wrung out in hot water), using hotter and hotter, until you come to the extreme heat. White corpuscles are young corpuscles; if they die, they become pus corpuscles.

Aconite stops neural circulation; so does heat. They drive back the circulation by contracting the capillaries. If pus corpuscles have formed, cut and let out these devils—*exorcise* them.

If abscess has opened, go through the root and enlarge it until blood comes through. Wash with water and tincture of calendula. Thus I get rid of all foreign substances, go through the alveolus, and amputate the end of the root if it is necrosed; fill with gold by making a shoulder inside of the pulp canal, and to this point in each root press the gold, filling it completely. Then open the fistula large enough to see the apex of the root, and cut or polish off, as you please. Inject sulphuric acid and water, taking care not to let it run over the mouth; then fill the opening with tannin and glycerin. Chloride of zinc may be used, 480 grs. to the oz. of distilled water. All fibrils found in teeth are post-mortem re sults. Tomes does not call them nerves.

A resolution authorizing committees to put on sale, at any depot, the printed copies of proceedings, was then carried, the price of the large books being fixed at $1.50, and the small at $1.00.

Adjourned.

---

